# The complete chloroplast genome of *Epimedium xichangense* Y. J. Zhang (Berberidaceae)

**DOI:** 10.1080/23802359.2020.1721353

**Published:** 2020-02-03

**Authors:** Yuanyue Wang, Xiang Liu, Cheng Zhang, Chaoqun Xu, Fengmei Suo, Guoan Shen, Baolin Guo

**Affiliations:** a Institute of Medicinal Plant Development, Chinese Academy of Medical Science, Peking Union Medical College, Beijing, China;; b Chongqing Academy of Chinese Materia Medica, Chongqing, China

**Keywords:** Chloroplast genome, *Epimedium xichangense*, Berberidaceae

## Abstract

*Epimedium xichangense*, a critically endangered herb with limited population, mainly distributes in Sichuan province, China. In our study, we obtained the complete chloroplast genome of *E. xichangense* with a length of 158,955 bp, including a large single copy region of 86,478 bp, small single copy region of 17,027 bp, and a pair of inverted repeat regions of 27,725 bp. The GC content in the whole chloroplast genome of *E. xichangense* is 38.81%. Among the 112 unique genes in the circular genome, 30 tRNA, four rRNA and 78 protein-coding genes were successfully annotated. We constructed the Maximum likelihood (ML) tree with 26 species, and came to the conclusion that *E. xichangense* was phylogenetically closely related to *E. acuminatum* and *E. chlorandrum*.

The *Epimedium* L., a perennial herbaceous genus of Berberidaceae family, is commonly known as a medicinal plant in the world. The therapeutic effects of Epimedii Folium include nourishing kidney, strengthening bones and relieving rheumatism, which had a long history using in traditional Chinese medicine (Ye and Chen 2001; Wu et al. 2003; Ma et al. 2011). *E. xichangense* is designated as a critically endangered species, which is only found in the Xichang County, Sichuan, China (Zhang et al. 2016). The chloroplast is an important organelle that has its own genomes, and the chloroplast genome of plants has been a focus of research in plant molecular evolution and systematics (Clegg et al. 1994). So far, six complete chloroplast genomes of *Epimedium* have been reported (Zhang et al. 2016; Liu et al. 2019). In this study, we sequenced chloroplast genes of *E. xichangense,* which is a valuable resource for further studies of the Berberidaceae family especially in terms of genetic evolution.

The fresh leaves were collected from the Xichang County, Sichuan, China (N27°53′, E102°15′). The voucher specimen (Guo0619) were deposited at the Herbarium of the Institute of Medicinal Plant (IMPLAD), Beijing, China. Genomic DNA was extracted using the modified CTAB method (Doyle and Doyle 1987). The sequencing was carried out on the Illumina Novaseq PE150 platform (Illumina Inc, San Diego), and 150 bp paired-end reads were generated. The software GetOrganelle v1.5 (Jin et al. 2018) was used to assemble the cleaned reads into a complete chloroplast genome, with *E. acuminatum* (GenBank accession number: NC_029941) chloroplast genome as a reference. The chloroplast genome annotation was performed through the online program CPGAVAS2 (Shi et al. 2019) and GeSeq (Tillich et al. 2017), followed by manual correction. The assembled chloroplast genome sequence has been submitted to GenBank with the accession number MN883539.

In the present study, the complete chloroplast genome of *E. xichangense* is 158,955 bp in length, which is a typical circular structure consisting of two reverse repeat regions (IRa and IRb) of 27,725 bp that separated by a large single copy (LSC, 86,478 bp) and a small single copy (SSC, 17,027 bp). The GC content in IR, LSC and SSC regions is 43.02%, 37.30% and 32.79%, respectively. The chloroplast genome was identified to have a total of 131 genes, including 85 protein-coding genes, 38 tRNA genes, and eight rRNA genes. Seven protein-coding genes (*rps*12, *ndhB*, *rps*7, *rpl*23, *rpl*2, *rps*19and *ycf*2), seven tRNA (*trnA-UGC*, *trnI-CAU*, *trnI-GAU*, *trnL-CAA*, *trnN-GUU*, *trnV-GAC*, and *trnR-ACG*), four rRNA (*rrn*16, *rrn*23, *rrn*4.5, and *rrn*5) appear twice in the inverted orientation, and one tRNA gene (*trnQ-UUG*) is duplicated in the LSC. Nine protein-coding genes (*ndhA*, *rps*16, *atpF*, *rpoC*1, *petB*, *petD*, *rpl*16, *rpl*2, and *ndhB*) and six tRNA genes (*trnK-UUU*, *trnG-UCC*, *trnL-UAA*, *trnV-UAC*, *trnI-GAU*, and *trnA-UGC*) contain one intron, and while three genes (*clpP*, *rps*12, and *ycf*3) contain two introns.

The chloroplast genomes of 23 species from Berberidaceae and as well as *Urophysa henryihernia* and *Helleborus thibetanus* as outgroup species were downloaded from the NCBI GenBank database to identify the phylogenetic relationship of *E. xichangense*. The sequences were aligned using MAFFT v7 (Katoh et al. 2019). In addition, a Maximum likelihood (ML) tree based on the common protein-coding genes of 26 species was constructed by using raxmlGUI1.5b (v8.2.10) (Silvestro and Michalak 2012). Phylogenetic analysis shows that *E. acuminatum* and *E. chlorandrum* are closely related to *E. xichangense* (Figure 1). This study will provide important information for species identification, chloroplast genetic engineering and phylogenetic relationship in Berberidaceae family.

**Figure 1.**
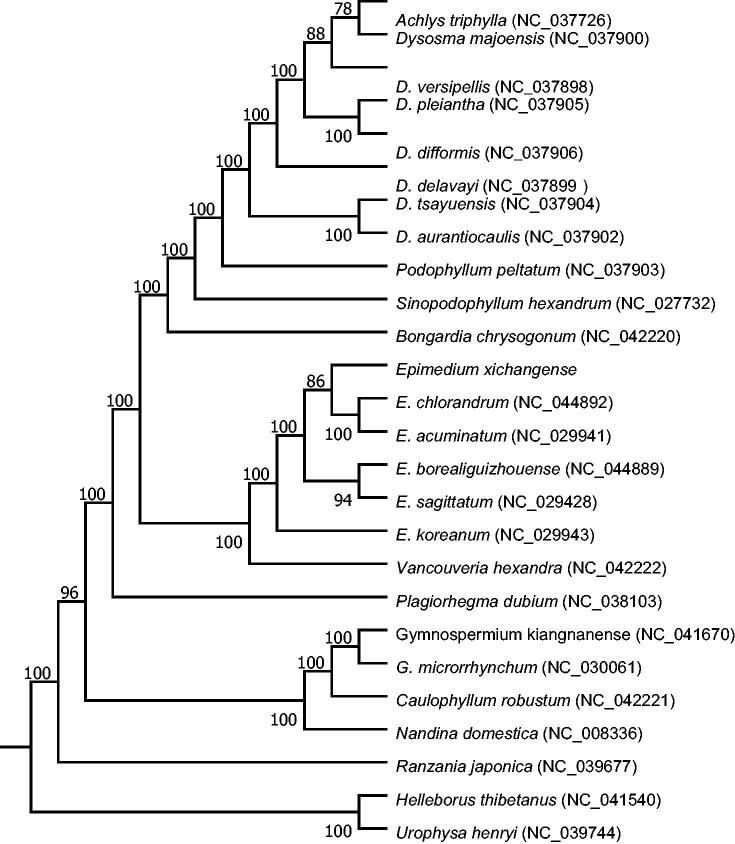
Phylogenetic tree reconstructed using maximum likelihood (ML) method based on the common protein-coding genes of 26 species, with *Urophysa henryihernia* and *Helleborus thibetanus* as the outgroup. Numbers above the lines represent ML bootstrap values (>70%).

## References

[CIT0001] Clegg M, Gaut B, Learn G, Morton B. 1994. Rates and patterns of chloroplast DNA evolution. Proc Natl Acad Sci. 91(15):6795–6801. 8041699 10.1073/pnas.91.15.6795 PMC44285

[CIT0002] Doyle J, Doyle J. 1987. A rapid DNA isolation procedure for small quantities of fresh leaf tissue. Phytochem Bull. 19:11–15.

[CIT0003] Jin J, Yu W, Yang J, Song Y, Yi T, Li D. 2018. GetOrganelle: a simple and fast pipeline for de novo assembly of a complete circular chloroplast genome using genome skimming data. bioRxiv. 4:256479.

[CIT0004] Katoh K, Rozewicki J, Yamada K. 2019. MAFFT online service: multiple sequence alignment, interactive sequence choice and visualization. Brief Bioinform. 20(4):1160–1166. 28968734 10.1093/bib/bbx108 PMC6781576

[CIT0005] Liu X, Yang Q, Zhang C, Shen G, Guo B. 2019. The complete chloroplast genome of *Epimedium sagittatum* (Sieb. Et Zucc.) Maxim. (Berberidaceae), a traditional Chinese herb. Mitochondrial DNA Part B. 4(2):2572–2573. 33365631 10.1080/23802359.2019.1640087 PMC7706818

[CIT0006] Ma H, He X, Yang Y, Li M, Hao D, Jia Z. 2011. The genus *Epimedium*: an ethnopharmacological and phytochemical review. J Ethnopharmacol. 134(3):519–541. 21215308 10.1016/j.jep.2011.01.001

[CIT0007] Shi L, Chen H, Jiang M, Wang L, Wu X, Huang L, Liu C. 2019. CPGAVAS2, an integrated plastome sequence annotator and analyser. Nucleic Acids Res. 47:65–73. 10.1093/nar/gkz345 PMC6602467 31066451

[CIT0008] Silvestro D, Michalak I. 2012. raxmlGUI: a graphical front-end for RAxML. Org Divers Evol. 12(4):335–337.

[CIT0009] Tillich M, Lehwark P, Pellizzer T, Ulbricht-Jones E, Fischer A, Bock R, Greiner S. 2017. GeSeq - versatile and accurate annotation of organelle genomes. Nucleic Acids Res. 45(W1):W6–W11. 28486635 10.1093/nar/gkx391 PMC5570176

[CIT0010] Wu H, Lien E, Lien L. 2003. Chemical and pharmacological investigations of Epimedium species: a survey. Prog Drug Res. 60(60):1–57. 12790338 10.1007/978-3-0348-8012-1_1

[CIT0011] Ye L, Chen J. 2001. Advances in study on pharmacological effects of Epimedium. CJCMM. 26(5):293–295. 12528515

[CIT0012] Zhang Y, Du L, Liu A, Chen J, Wu L, Hu W, Zhang W, Kim K, Lee S, Yang T, et al. 2016. The complete chloroplast genome sequences of five Epimedium species: lights into phylogenetic and taxonomic analyses. Front Plant Sci. 7:306 27014326 10.3389/fpls.2016.00306 PMC4791396

[CIT0013] Zhang Y, Zhang S, Dang H, Zhang B, Li J, Wang Y. 2016. *Epimedium xichangense* (Berberidaceae), a new species from Sichuan, China. Phytotaxa. 263(3):286–290.

